# Serum Immunoglobulin G Levels to *Porphyromonas gingivalis* Peptidylarginine Deiminase Affect Clinical Response to Biological Disease-Modifying Antirheumatic Drug in Rheumatoid Arthritis

**DOI:** 10.1371/journal.pone.0154182

**Published:** 2016-04-25

**Authors:** Tetsuo Kobayashi, Satoshi Ito, Daisuke Kobayashi, Atsushi Shimada, Ichiei Narita, Akira Murasawa, Kiyoshi Nakazono, Hiromasa Yoshie

**Affiliations:** 1 General Dentistry and Clinical Education Unit, Niigata University Medical and Dental Hospital, Niigata, Japan; 2 Division of Periodontology, Department of Oral Biological Science, Niigata University Graduate School of Medical and Dental Sciences, Niigata, Japan; 3 Department of Rheumatology, Niigata Rheumatic Center, Shibata, Japan; 4 Division of Clinical Nephrology and Rheumatology, Department of Homeostatic Regulation Developments, Niigata University Graduate School of Medical and Dental Sciences, Niigata, Japan; University of Leuven, Rega Institute, BELGIUM

## Abstract

**Objectives:**

To determine whether serum immunity to *Porphyromonas gingivalis* peptidylarginine deiminase (PPAD) affects the clinical response to biological disease-modifying antirheumatic drug (bDMARD) in patients with rheumatoid arthritis (RA).

**Methods:**

In a retrospective study, rheumatologic and periodontal conditions of 60 patients with RA who had been treated with conventional synthetic DMARD were evaluated before (baseline) and after 3 and 6 months of bDMARD therapy. After serum levels of anti-PPAD immunoglobulin G (IgG) were determined at baseline, the patients were respectively divided into two groups for high and low anti-PPAD IgG titers according to the median measurements. Genotypes at 8 functional single nucleotide polymorphisms (SNPs) related to RA were also determined.

**Results:**

After 3 and 6 months of therapy, patients with low anti-PPAD IgG titers showed a significantly greater decrease in changes in the Disease Activity Score including 28 joints using C-reactive protein (DAS28-CRP) (P = 0.04 for both) and anti-cyclic citrullinated peptide (CCP) IgG levels (P = 0.03 and P = 0.04) than patients with high anti-PPAD IgG titers, although these parameter values were comparable at baseline. The anti-PPAD IgG titers were significantly positively correlated with changes in the DAS28-CRP (P = 0.01 for both) and the anti-CCP IgG levels (P = 0.02 for both) from baseline to 3 and 6 months later. A multiple regression analysis revealed a significantly positive association between the anti-PPAD IgG titers and changes in the DAS28-CRP after 6 months of bDMARD therapy (P = 0.006), after adjusting for age, gender, smoking, periodontal condition, and RA-related SNPs.

**Conclusion:**

The serum IgG levels to PPAD affect the clinical response to bDMARD in patients with RA.

## Introduction

Rheumatoid arthritis (RA) is a systemic autoimmune disease that encompasses a breach of self-tolerance, chronic synovial inflammation and joint destruction [1]. Periodontitis is also a chronic inflammatory disease characterized by local inflammation and destruction of the periodontal tissue. RA and periodontitis exhibit similar pathological features that are associated with the overproduction of pro-inflammatory cytokines such as interleukin-6 (IL-6) and tumor necrosis factor-alpha (TNF-α) [2,3]. The serum levels of IL-6 and TNF-α were increased in the patients with RA in comparison to normal controls [4]. Patients with periodontitis also displayed higher serum levels of IL-6 and TNF-α than the periodontally healthy individuals [5]. A beneficial effect of treatment with inhibitors of TNF and IL-6 receptor (TNFI and IL-6RI) has been suggested on periodontal inflammation as well as the rheumatologic condition in patients with RA [2,6]. These observations imply a potential non-causal association between RA and periodontitis [7].

As a plausible causal mechanism, there is increasing evidence to suggest that, among periodontopathogens, *Porphyromonas gingivalis* (*P*. *gingivalis*) plays an important role in the production of anti-cyclic citrullinated peptide (CCP) antibodies and the etiology of RA [7–9]. *P*. *gingivalis* peptidylarginine deiminase (PPAD) is an enzyme that modifies peptidylarginine residues to citrulline [10]. Recent evidences indicated that serum immunoglobulin G (IgG) responses to PPAD were elevated in patients with RA compared with patients with periodontitis and healthy individuals [11,12]. Experimental arthritis studies have also demonstrated that a PAD-deficient strain of *P*. *gingivalis* was associated with a reduced serum response to CCP [13,14]. These findings suggest the association between anti-PPAD and anti-CCP antibody responses, but are different from the results of another study [15]. Therefore, it is of clinical importance to determine whether serum anti-PPAD immunity affects protein citrullination and the onset and progression of RA.

Studies suggest that serum anti-CCP antibody responses correlate with the disease severity of RA and is a sensitive and specific marker for the onset and disease progression of RA [16,17]. A number of clinical trials have demonstrated a decrease in the disease activity after treatment with biological disease-modifying antirheumatic drug (bDMARD) including TNFI and IL-6RI [18–20]. Additionally, these clinical responses to bDMARD were affected by serum levels of anti-CCP antibodies [21,22]. These observations lead to the hypothesis that elevated serum IgG levels to PPAD may result in a poor clinical response to bDMARD by regulating anti-CCP immunity. However, to date, no study has evaluated the anti-PPAD IgG titers and clinical response to bDMARD.

Therefore, the aim of the present study was to assess whether serum anti-PPAD IgG titers affect the clinical response to bDMARD and correlate to the autoantibodies in patients with RA.

## Methods

### Ethics statement

The present study was conducted in accordance with the Declaration of Helsinki and was approved by the Institutional Review Board of the Niigata University Faculty of Dentistry (Permit Number 23-R2-11-05, 2011) and Niigata Rheumatic Center (Permit Number 2, 2011). All participants provided their written informed consent to participate in the present study.

### Study design

A retrospective cohort study was conducted at Niigata Rheumatic Center in conjunction with the Division of Periodontology, Department of Oral Biological Science, Niigata University Graduate School of Medical and Dental Sciences. Inclusion criteria were patients who were diagnosed with RA according to the 2010 RA classification criteria of the American College of Rheumatology and European League Against Rheumatism (EULAR) [1], those who had been treated with conventional synthetic DMARD (csDMARD) before they entered the study, and those who were treated with bDMARD including TNFI and IL-6RI between July 2011 and January 2015. Exclusion criteria were patients who were diagnosed with diabetes mellitus with HbA1c ≥ 6.5% and fasting plasma glucose ≥ 126 mg/dl [23], those who were pregnant, those who had received antibiotic and periodontal treatments within the last 3 months, and those whose number of teeth present was fewer than 15. The study schedule consisted of rheumatologic and periodontal assessments and blood collections at baseline (pre-treatment) and 3 and 6 months of the first administration of bDMARD including TNFI and IL-6RI. Rheumatologists and periodontists evaluated each patient independently and were blinded from each other regarding the rheumatologic and periodontal condition. None of the patients received any tooth-brushing instructions or periodontal treatments and were instructed not to change their oral hygiene regimens throughout the study period.

### Rheumatologic assessment

Clinical rheumatologic data were obtained by the rheumatologists (SI, DK, AM, KN) and stored in the Niigata Rheumatic Center database, which included the disease duration and activity of RA, tender and swollen joint counts, and the patient’s general assessment of his/her condition scored on a visual analog scale (VAS). The disease activity of RA was determined in all patients with the Disease Activity Score including 28 joints using C-reactive protein (DAS28-CRP), which was calculated based on four components: tender joint count, swollen joint count, VAS, and CRP measurements [24]. Serological rheumatologic data included anti-CCP IgG titers that were determined by an enzyme-linked immunosorbent assay (ELISA) with a commercially available kit (Medical & Biological Laboratories, Co., Ltd. Aichi, Japan) [25], and the serum levels of rheumatoid factor (RF) and CRP that were measured with latex particle-enhanced and simple nephelometric methods (SRL, Tokyo, Japan) [12], respectively. Positivity for RF and anti-CCP IgG were defined as measurements more than 15 IU/mL and 4.5 U/mL, respectively.

### Periodontal assessment

Clinical periodontal data were collected by one calibrated periodontist (TK) as previously described [12], which included the number of teeth present, O'Leary's plaque control record [26], gingival index [27], bleeding on probing, probing depth, and clinical attachment level. The bleeding on probing, probing depth and clinical attachment level were measured at six sites around each tooth with a Williams probe (Hu-Friedy, Chicago, IL, USA). The definition of periodontitis was based on the classification criteria of the 5th European Workshop in Periodontology, which involves two categories of periodontitis [28]. A moderate case was defined by the presence of proximal attachment loss of ≥ 3 mm in ≥ 2 non-adjacent teeth, while a severe case was defined by the presence of proximal attachment loss of ≥ 5 mm in ≥ 30% of teeth present [28]. The average score for full-mouth bleeding on probing was expressed as the percentage of sites with bleeding in the total number of sites examined in each patient. The average score for full-mouth probing depth and clinical attachment level was also calculated in each patient. The smoking status, which can affect the periodontal condition, was recorded by a standard questionnaire.

### Determination of serum anti-PPAD titer

Serum levels of anti-PPAD IgG were determined by an ELISA as previously described [12], and expressed as ELISA units (EU). The PPAD peptide sequence CLGTDALHC-Cit-THEVADKGC was used for the ELISA [11].

### SNPs analyses

Genomic DNA was extracted from blood samples using a DNA extractor kit (Wako Pure Chemical Industries, Osaka, Japan) according to the manufacturer’s instructions. A total of 8 SNPs were selected according to documented associations with RA or responses to bDMARD therapy (rs2240340, rs2240337, and rs1748033 for PADI4, rs33996649 and rs2476601 for PTPN22, rs1800630 and rs1799724 for TNFA and rs1800796 for IL-6) [29–32]. Genotypes at these SNPs were determined with a nano-Invader DNA chip system [33]. Genotyping was performed four times in each sample, and the consistent rate for each SNP was more than 99%.

### Statistical analyses

Prior to the present study, the sample size calculation was performed with a parametric test using the SPSS software program (SamplePower version 3.0, IBM, Chicago, IL, USA) with serum anti-PPAD IgG titers that were shown in a previous study [12]. The results revealed that more than 24 patients in each of the two groups exceeded a statistical power of 0.8 with an alpha level of 5% and an anticipated effect size of 0.8. Differences in quantitative data between the two groups were assessed by Mann Whitney *U*-tests, after evaluating the normality of distribution by Kolmogorov-Smirnov tests. Chi-square and Fisher's exact tests were used to compare categorical data between the two groups. Spearman’s rank correlation coefficient was used to determine the relationship between the serum anti-PPAD IgG titers and changes in the DAS28-CRP and anti-CCP IgG levels. A multiple regression analysis was performed to assess the association between the serum anti-PPAD IgG titers and changes in the DAS28-CRP, after adjusting for age, gender, smoking, periodontal condition, and RA-related SNPs, using a statistical software program (SPSS statistics version 21, IBM Japan, Tokyo, Japan). Confounders considered for this multiple regression analysis included patient age, gender (male = 0, female = 1), smoking status (never-smoker = 0, former-smoker = 1, current-smoker = 2), and SNP (homozygous wild type = 0, heterozygous type = 1, homozygous mutant type = 2). Statistical significance was considered to exist at 5% (P < 0.05).

## Results

### Patient baseline characteristics

The baseline characteristics of the 60 patients are shown in Table 1. After determination of the baseline serum anti-PPAD IgG titers by an ELISA, 30 patients whose anti-PPAD IgG titers were higher than the median measurements (0.822 EU) were selected for the high titer group; another 30 patients whose anti-PPAD IgG titers were lower than the median were selected for the low titer group. The high titer group displayed significantly higher serum IgG levels to PPAD than the low titer group ([mean ± SD] 1.7 ± 0.6 EU versus 0.6 ± 0.2 EU, P < 0.001). No significant differences were observed at baseline between the groups in any demographic, periodontal, and rheumatologic parameter values (P > 0.05) (Table 1). The prevalence of previous and current RA medications including corticosteroid, conventional synthetic DMARD (csDMARD), and non-steroidal anti-inflammatory drug (NSAID) was not significantly different between the groups (P > 0.05) (Table 1). The proportion of patients who were treated with TNFI and IL-6RI was comparable between the groups (76.7% versus 86.7% for TNFI, 23.3% versus 13.3% for IL-6RI, P > 0.05 for both comparisons) (Table 1). A similar proportion of each TNFI in the anti-TNF therapy was also confirmed between the groups ([high and low titer group]: 7 and 5 cases for infliximab, 6 and 3 cases for etanercept, 6 and 15 cases for adalimumab, 3 and 2 cases for golimumab, and 1 and 1 cases for certolizumab pegol, respectively) (P > 0.05).

**Table 1:** Baseline characteristics of the study patients with rheumatoid arthritis (RA).

Parameter	All patients (N = 60)	High titer group ^a^ (N = 30)	Low titer group ^a^ (N = 30)	Intergroup P-value
Demographic				
Age, mean (SD) years	55.7 (11.4)	55.1 (11.6)	56.4 (11.4)	0.80
Female, no. (%)	54 (90.0)	27 (90.0)	27 (90.0)	0.99
Smoker of current/former/never, %	0/15/85	0/13/87	0/17/83	0.72
Periodontal				
Teeth present, no. (SD)	25.0 (4.1)	24.6 (4.7)	25.4 (3.5)	0.52
Plaque control record, mean (SD) %	36.2 (21.1)	39.3 (22.9)	33.2 (19.2)	0.33
Gingival index, mean (SD)	1.0 (0.2)	1.0 (0.2)	1.0 (0.2)	0.68
Bleeding on probing, mean (SD) %	9.7 (10.9)	12.1 (13.7)	7.2 (6.5)	0.20
Probing depth, mean (SD) mm	2.6 (0.3)	2.6 (0.4)	2.6 (0.3)	0.85
Probing depth ≥ 4mm, mean (SD) %	11.2 (13.0)	13.6 (15.1)	8.8 (10.3)	0.21
Clinical attachment level, mean (SD) mm	2.7 (0.3)	2.7 (0.3)	2.7 (0.3)	0.80
Clinical atatchment level ≥ 4mm, mean (SD) %	12.8 (13.6)	14.8 (14.8)	10.7 (12.2)	0.25
Moderate periodontitis ^b^, no. (%)	20 (33.3)	11 (36.7)	9 (30.0)	0.59
Severe periodontitis ^b^, no. (%)	2 (3.3)	1 (3.3)	1 (3.3)	0.99
Rheumatologic				
Disease duration, mean (SD) months	78.1 (75.6)	77.8 (71.5)	78.4 (80.7)	0.68
DAS28-CRP, mean (SD)	3.9 (1.1)	4.0 (1.3)	3.9 (1.0)	0.56
Remission/low/moderate/high activity (%)	6/6/50/37	3/10/54/33	10/3/47/40	0.72
Tender joint count, mean (SD)	5.0 (5.3)	4.8 (5.9)	5.1 (4.7)	0.43
Swollen joint count, mean (SD)	4.4 (4.6)	5.0 (5.4)	3.9 (3.6)	0.70
VAS, mean (SD) mm	42.7 (21.3)	44.3 (20.2)	41.1 (22.6)	0.75
Corticosteroid use, no. (%)	40 (66.7)	21 (70.0)	19 (63.3)	0.59
csDMARD use, no. (%)	57 (95.0)	30 (100.0)	27 (90.0)	0.08
NSAID use, no (%)	23 (38.3)	11 (36.7)	12 (40.0)	0.79
TNF inhibitor use, no. (%)	49 (81.7)	23 (76.7)	26 (86.7)	0.32
IL-6R inhibitor use, no. (%)	11 (18.3)	7 (23.3)	4 (13.3)	0.32
Anti-CCP IgG level, mean (SD) U/mL	134.6 (148.5)	132.7 (168.5)	136.5 (128.3)	0.33
Anti-CCP IgG positive, no. (%)	50 (83.3)	23 (76.7)	27 (90.0)	0.17
RF level, mean (SD) IU/mL	151.7 (291.1)	181.6 (381.8)	121.8 (157.4)	0.87
RF positive, no. (%)	53 (88.3)	26 (86.7)	27 (90.0)	0.69
Laboratory				
CRP level, mean (SD) mg/dL	2.4 (2.6)	3.1 (3.2)	1.8 (1.7)	0.29
Anti-PPAD IgG level, mean (SD) EU	1.1 (0.7)	1.7 (0.6)	0.6 (0.2)	< 0.001*

SD: standard deviation; DAS28-CRP: disease activity score including 28 joints using C-reactive protein; VAS: visual analog scale; csDMARD: conventional synthetic disease-modifying antirheumatic drug; NSAID: non-steroidal anti-inflammatory drug; TNF: tumor necrosis factor; IL-6R: interleukin-6 receptor; CCP: cyclic citrullinated peptide; IgG: immunoglobulin G; RF: rheumatoid factor; PPAD: *Porphyromonas gingivalis* peptidylarginine deiminase; EU: ELISA unit.

^a^ Anti-PPAD IgG cut-off level, 0.822 EU.

^b^ Moderate and severe periodontitis was defined according to the classification criteria of the 5th European Workshop in Periodontology (28).

* Significantly different between the groups (P < 0.05) (bold value).

### Anti-PPAD IgG titers and the rheumatologic condition

**Fig 1:**
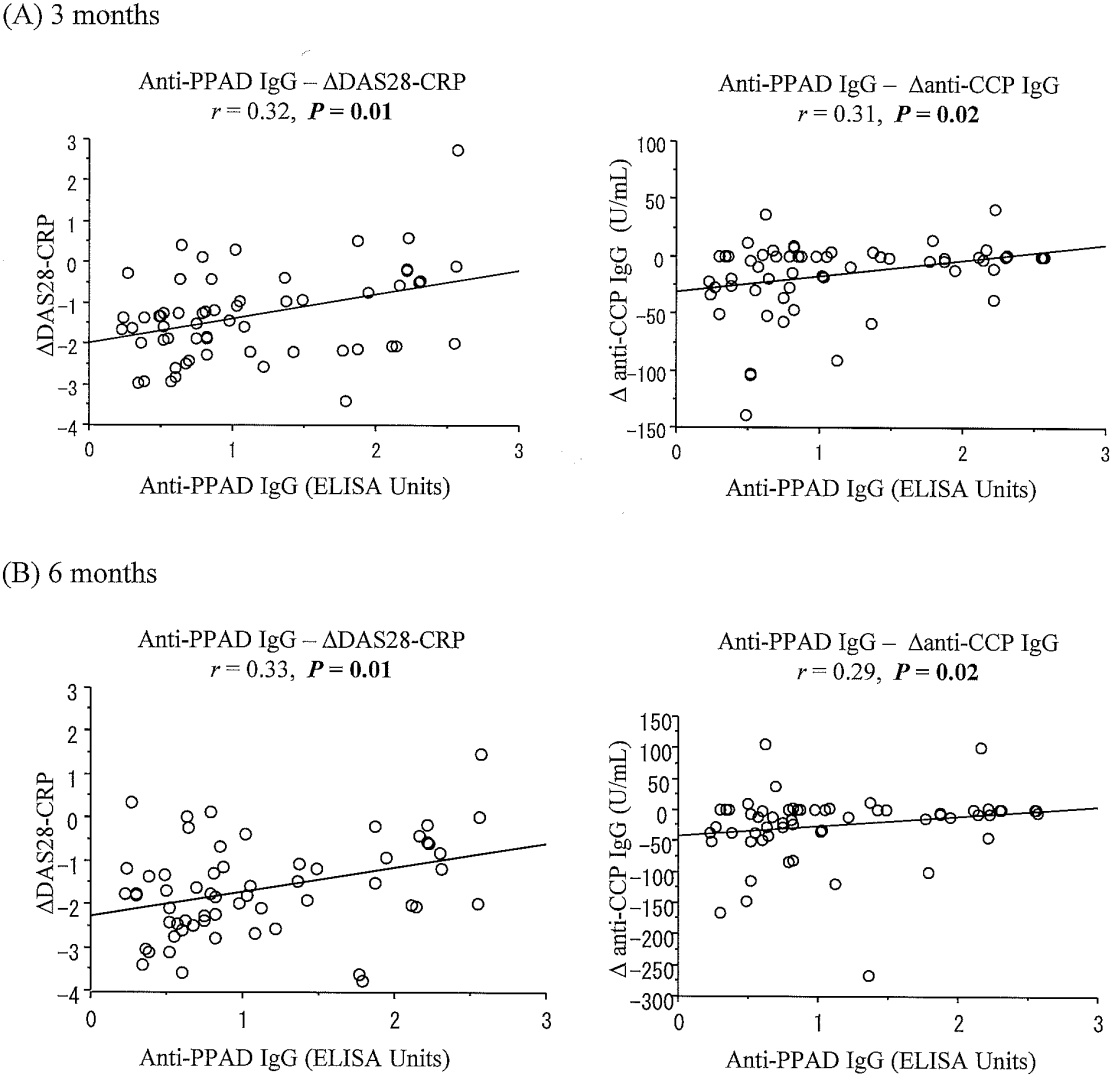
Relevance of the serum anti-PPAD IgG levels to changes in the DAS28-CRP and serum anti-CCP IgG levels after 3 and 6 months of bDMARD therapy (A and B).

The low titer group showed significantly lower scores in the DAS28-CRP than the high titer group both at 3 and 6 months later (2.2 ± 0.7 versus 2.9 ± 1.1, P = 0.02 at 3 months; 2.0 ± 0.6 versus 2.5 ± 0.8, P = 0.005 at 6 months) (Tables 2 and 3). Other rheumatologic and periodontal parameter values were comparable between the groups at 3 and 6 months later (P > 0.05) (Tables 2 and 3). A significantly greater decrease was also observed in changes in the DAS28-CRP from baseline to 3 and 6 months later in the low titer group than in the high titer group (-1.7 ± 0.9 versus -1.0 ± 1.2, P = 0.04 at 3 months; -1.9 ± 1.0 versus -1.4 ± 1.1, P = 0.04 at 6 months) (Table 4). Likewise, a greater decrease was found in changes in the anti-CCP IgG levels from baseline to 3 and 6 months later in the low titer group than in the high titer group (-25.5 ± 37.8 U/mL versus -6.4 ± 22.7 U/mL, P = 0.03 at 3 months; -31.3 ± 52.2 U/mL versus -19.2 ± 58.7 U/mL, P = 0.04 at 6 months) (Table 4). The low titer group exhibited a significantly greater decrease in the tender joint count than the high titer group at 3 months later (-3.8 ± 4.0 versus -1.9 ± 5.1, P = 0.01) (Table 4), but not at 6 months. Furthermore, the anti-PPAD IgG titers were significantly positively correlated with changes in the DAS28-CRP and anti-CCP IgG levels both at 3 and 6 months later (P = 0.01 and P = 0.02 for both) (Fig 1A and 1B).

**Table 2:** Comparison of characteristics after 3 months of biological disease-modifying antirheumatic drug (bDMARD) therapy between the patients with high and low anti-*Porphyromonas gingivalis* peptidylarginine deiminase (PPAD) immunoglobulin G (IgG) titers.

Parameter	High titer group ^a^ (N = 30)	Low titer group ^a^ (N = 30)	P-value
Periodontal			
Teeth present, no. (SD)	24.6 (4.7)	25.4 (3.5)	0.52
Plaque control record, mean (SD) %	33.4 (19.7)	32.0 (20.9)	0.67
Gingival index, mean (SD)	0.8 (0.2)	0.9 (0.2)	0.26
Bleeding on probing, mean (SD) %	6.6 (9.4)	5.3 (8.0)	0.61
Probing depth, mean (SD) mm	2.5 (0.3)	2.5 (0.3)	0.99
Clinical attachment level, mean (SD) mm	2.7 (0.5)	2.7 (0.4)	0.95
Rheumatologic			
DAS28-CRP, mean (SD)	2.9 (1.1)	2.2 (0.7)	0.02*
Tender joint count, mean (SD)	3.0 (3.7)	1.3 (1.4)	0.07
Swollen joint count, mean (SD)	2.9 (3.7)	1.4 (2.0)	0.10
VAS, mean (SD) mm	28.9 (19.6)	20.4 (18.3)	0.06
Anti-CCP IgG level, mean (SD) U/mL	126.2 (166.8)	111.0 (126.7)	0.57
RF level, mean (SD) IU/mL	155.8 (375.9)	79.0 (122.9)	0.41
CRP level, mean (SD) mg/dL	0.8 (1.5)	0.1 (0.2)	0.05

SD: standard deviation; DAS28-CRP: disease activity score including 28 joints using C-reactive protein; VAS: visual analog scale; CCP: cyclic citrullinated peptide; RF: rheumatoid factor.

^a^ Anti-PPAD IgG cut-off level, 0.822 ELISA unit.

* Significantly different between the groups (P < 0.05) (bold value).

**Table 3:** Comparison of characteristics after 6 months of biological disease-modifying antirheumatic drug (bDMARD) therapy between the patients with high and low anti-*Porphyromonas gingivalis* peptidylarginine deiminase (PPAD) immunoglobulin G (IgG) titers.

Parameter	High titer group ^a^ (N = 30)	Low titer group ^a^ (N = 30)	P-value
Periodontal			
Teeth present, no. (SD)	24.6 (4.7)	25.4 (3.5)	0.52
Plaque control record, mean (SD) %	30.1 (17.8)	30.3 (22.5)	0.78
Gingival index, mean (SD)	0.8 (0.2)	0.8 (0.2)	0.46
Bleeding on probing, mean (SD) %	6.0 (7.4)	4.2 (6.5)	0.31
Probing depth, mean (SD) mm	2.5 (0.3)	2.5 (0.3)	0.88
Clinical attachment level, mean (SD) mm	2.7 (0.5)	2.6 (0.4)	0.83
Rheumatologic			
DAS28-CRP, mean (SD)	2.5 (0.8)	2.0 (0.6)	0.005*
Tender joint count, mean (SD)	1.8 (2.4)	1.1 (1.1)	0.82
Swollen joint count, mean (SD)	1.5 (2.0)	1.2 (2.1)	0.36
VAS, mean (SD) mm	19.1 (15.4)	24.2 (36.9)	0.85
Anti-CCP IgG level, mean (SD) U/mL	113.5 (162.9)	105.2 (129.9)	0.49
RF level, mean (SD) IU/mL	109.6 (199.7)	66.2 (93.7)	0.34
CRP level, mean (SD) mg/dL	0.4 (0.7)	0.2 (0.4)	0.39

SD: standard deviation; DAS28-CRP: disease activity score including 28 joints using C-reactive protein; VAS: visual analog scale; CCP: cyclic citrullinated peptide; RF: rheumatoid factor.

^a^ Anti-PPAD IgG cut-off level, 0.822 ELISA unit.

* Significantly different between the groups (P < 0.05) (bold value).

**Table 4:** Comparison of changes in rheumatologic conditions between the patients with high and low anti-*Porphyromonas gingivalis* peptidylarginine deiminase (PPAD) immunoglobulin G (IgG) titers.

Parameter	High titer group ^a^ (N = 30)	Low titer group ^a^ (N = 30)	P-value
3 months changes in ^b^			
DAS28-CRP, mean (SD)	-1.0 (1.2)	-1.7 (0.9)	0.04*
Tender joint count, mean (SD)	-1.9 (5.1)	-3.8 (4.0)	0.01*
Swollen joint count, mean (SD)	-2.0 (5.7)	-2.5 (3.5)	0.79
VAS, mean (SD) mm	-15.4 (19.9)	-20.7 (26.8)	0.51
Anti-CCP IgG level, mean (SD) U/mL	-6.4 (22.7)	-25.5 (37.8)	0.03*
RF level, mean (SD) IU/mL	-25.7 (61.8)	-42.8 (97.2)	0.27
6 months changes in ^b^			
DAS28-CRP, mean (SD)	-1.4 (1.1)	-1.9 (1.0)	0.04*
Tender joint count, mean (SD)	-3.1 (4.3)	-4.0 (4.5)	0.33
Swollen joint count, mean (SD)	-3.5 (5.1)	-2.7 (3.7)	0.66
VAS, mean (SD) mm	-25.2 (24.5)	-16.9 (45.4)	0.57
Anti-CCP IgG level, mean (SD) U/mL	-19.2 (58.7)	-31.3 (52.2)	0.04*
RF level, mean (SD) IU/mL	-72.0 (294.4)	-55.6 (107.0)	0.21

SD: standard deviation; DAS28-CRP: disease activity score including 28 joints using C-reactive protein; VAS: visual analog scale; CCP: cyclic citrullinated peptide; RF: rheumatoid factor.

^a^ Anti-PPAD IgG cut-off level, 0.822 ELISA unit.

^b^ Changes from baseline (-: decrease and +: increase).

* Significantly different between the groups (P < 0.05) (bold value).

The genotype and allele frequencies of the 8 polymorphisms (PADI4_94, PADI4_102, PADI4_104, PTPN22 R263Q, PTPN22 R620W, TNFA-863, TNFA -857, and IL-6–572) in relation to RA or response to bDMARD are shown in Table 5. No significant differences were observed in any genotype or allele frequencies between the high and low titer groups (P > 0.05) (Table 5).

**Table 5:** Comparison of genotype and allele frequencies of 8 polymorphisms between the patients with high and low anti-*Porphyromonas gingivalis* peptidylarginine deiminase (PPAD) immunoglobulin G (IgG) titers.

Chromosome	Polymorphism	Genotype Allele	High titer group ^a^ (N = 30)	Low titer group ^a^ (N = 30)	P-value
1p36	PADI4_94	C/C	8 (26.7)	6 (20.0)	0.21
	rs2240340	C/T	17 (56.7)	13 (43.3)	
		T/T	5 (16.7)	11 (36.7)	
		C	33 (55.0)	25 (41.7)	0.20
		T	27 (45.0)	35 (58.3)	
1p36	PADI4_102	C/C	27 (90.0)	23 (76.7)	0.31
	rs2240337	C/T	3 (10.0)	6 (20.0)	
		T/T	0 (0.0)	1 (3.3)	
		C	57 (95.0)	52 (86.7)	0.20
		T	3 (5.0)	8 (13.3)	
1p36	PADI4_104	C/C	8 (26.7)	8 (26.7)	0.17
	rs1748033	C/T	17 (56.7)	11 (36.7)	
		T/T	5 (16.7)	11 (36.7)	
		C	33 (55.0)	27 (45.0)	0.36
		T	27 (45.0)	33 (55.0)	
1p13.2	PTPN22 R263Q	G/G	30 (100.0)	30 (100.0)	N.D.
	rs33996649	G/A	0 (0.0)	0 (0.0)	
		A/A	0 (0.0)	0 (0.0)	
		G	60 (100.0)	60 (100.0)	N.D.
		A	0 (0.0)	0 (0.0)	
1p13.2	PTPN22 R620W	C/C	30 (100.0)	30 (100.0)	N.D.
	rs2476601	C/T	0 (0.0)	0 (0.0)	
		T/T	0 (0.0)	0 (0.0)	
		C	60 (100.0)	60 (100.0)	N.D.
		T	0 (0.0)	0 (0.0)	
6p21.3	TNFA -863	C/C	23 (76.7)	25 (83.3)	0.35
	rs1800630	C/A	5 (16.7)	5 (16.7)	
		A/A	2 (6.7)	0 (0.0)	
		C	51 (85.0)	55 (91.7)	0.39
		A	9 (15.0)	5 (8.3)	
6p21.3	TNFA -857	C/C	19 (63.3)	20 (66.7)	0.79
	rs1799724	C/T	9 (30.0)	7 (23.3)	
		T/T	2 (6.7)	3 (10.0)	
		C	47 (78.3)	47 (78.3)	N.D.
		T	13 (21.7)	13 (21.7)	
7p21	IL-6–572	C/C	19 (63.3)	17 (56.7)	0.86
	rs1800796	C/G	10 (33.3)	12 (40.0)	
		G/G	1 (3.3)	1 (3.3)	
		C	48 (80.0)	46 (76.7)	0.83
		G	12 (20.0)	14 (23.3)	

Values represent the numbers (%).

N.D.: not determined.

^a^ Anti-PPAD IgG cut-off level, 0.822 ELISA unit.

A multiple regression analysis revealed a significant positive association between the anti-PPAD IgG titers and changes in the DAS28-CRP at 6 months later (P = 0.006, odds ratio = 0.39, 95% confidence interval = 0.18 to 1.00), after adjusting for age, gender, smoking, periodontal condition, and RA-related SNPs (Table 6).

**Table 6:** Significance of association between characteristics and changes in the disease activity after 6 months of biological disease-modifying antirheumatic drug (bDMARD) therapy.

Parameter for DAS28-CRP	OR (95% CI)	P-value
Age	-0.01 (-0.03 to 0.02)	0.97
Women	-0.01 (-1.13 to 1.09)	0.97
Former smoker	0.12 (-0.50 to 1.20)	0.41
Plaque control record	0.10 (-0.01 to 0.02)	0.53
Probing depth	-0.22 (-1.89 to 0.43)	0.21
Anti-PPAD IgG titer	0.39 (0.18 to 1.00)	0.006*
PADI4_94 SNP	0.05 (-1.05 to 1.18)	0.90
PADI4_102 SNP	0.30 (-0.63 to 1.48)	0.42
PADI4_104 SNP	-0.01 (-0.74 to 0.67)	0.92
PTPN22 R263Q SNP	N.D.	N.D.
PTPN22 R620W SNP	N.D.	N.D.
TNFA -863 SNP	-0.14 (-0.88 to 0.29)	0.31
TNFA -857 SNP	-0.12 (-0.66 to 0.26)	0.38
IL-6–572 SNP	0.16 (-0.20 to 0.82)	0.23

DAS28-CRP: disease activity score including 28 joints using C-reactive protein; PPAD: *Porphyromonas gingivalis* peptidylarginine; IgG: immunoglobulin G; TNF: tumor necrosis factor; IL-6: interleukin-6; OR: odds ratio; CI: confidence interval; N.D.: not determined.

* Significantly associated with changes in DAS28-CRP (P < 0.05) (bold value).

## Discussion

To the best of our knowledge, this is the first longitudinal study to evaluate the association between the baseline anti-PPAD IgG titers and clinical response to bDMARD therapy in patients with RA.

The results of univariate analyses showed a better improvement in the DAS28-CRP and anti-CCP IgG level in patients with low baseline anti-PPAD IgG titers in the age-, gender-, smoking status-, periodontal and rheumatologic condition-, and RA-related SNPs distribution-matched cohorts. Additionally, the data indicated that the baseline anti-PPAD IgG titers were positively correlated with changes in the DAS28-CRP and anti-CCP IgG levels, which is in agreement with the findings of previous studies [12,34–36]. However, these observations are different from the findings of another study [15], which might be partially explained by differences in the ELISA protocol [15], in the PPAD peptide sequence that is recognized by the anti-PPAD antibodies [37], or in the patient cohort studied. Furthermore, the results of the multiple regression analysis demonstrated that the baseline anti-PPAD IgG titers were positively associated with changes in the DAS28-CRP after adjusting for age, gender, smoking, periodontal condition, and RA-related SNPs. Another confounding factor, RA medication, was unchanged in all patients during the study period. These results suggest that low baseline anti-PPAD IgG titers are associated with a better clinical response to bDMARD therapy, which might be due to decreased protein citrullination in relation to the pathogenesis of RA.

A great effort has been made to identify serological and genetic markers to predict the clinical response to bDMARD including TNFI and IL-6RI. The presence of RF and anti-CCP antibodies were associated with anti-TNF responses [21,22], and a high baseline CRP level was a predictive factor for a better response to tocilizumab therapy [38,39]. Studies have also suggested the role of gene polymorphisms in the genes encoding TNF, IL-6, and PAD-4 that affect susceptibility to RA [29–31] and the response to TNFI including infliximab, etanercept, adalimumab, and certolizumab pegol [32]. However, in the present study, the clinical response to TNFI and IL-6RI, as determined by changes in the DAS28-CRP, tender and swollen joint counts, and VAS, was not influenced by the baseline serum levels of RF, anti-CCP IgG, and CRP, as well as by the genetic variants of 8 RA-related SNPs. These results suggest that the influence of the baseline anti-PPAD IgG titers on the efficacy of bDMARD might be independent of these serological and genetic factors related to RA. However, it might be necessary to further study other candidate genes such as Fcγ receptor IIIA and human leukocyte antigen-DRB1 in relation to the response to bDMARD therapy [40].

It was interesting to find a significant difference in the baseline anti-PPAD IgG titers of the groups despite their similar rheumatological and periodontal parameters. It is well recognized that periodontitis is caused by a persistent polymicrobial infection, and that *P*. *gingivalis* does not have to be present in patients with periodontitis [34, 41]. Thus, the difference in the anti-PPAD IgG titers might be partially explained by a difference in the frequency of *P*. *gingivalis*-associated periodontitis in each of the groups. Additionally, it has been reported that the severity of periodontitis was related to RA disease activity [42], which suggests a link between the rheumatological and periodontal parameter values. Furthermore, the results indicated no difference in the periodontal conditions of the groups, which implies that the anti-PPAD IgG titers were not correlated with the severity of periodontitis. These results were in accordance with the findings of a previous study [12], and were supported by the observations of another study that exhibited a borderline association between the serum antibody levels to *P*. *gingivalis* and the number of teeth with periodontal pockets of ≥ 4 mm in depth [43].

There are some methodological limitations associated with the present study. First, the sample size was relatively small due to the strict inclusion and exclusion criteria. In particular, patients with high RA activity that had been initiated with bDMARD therapy were excluded in the present study. Therefore, it was difficult to evaluate the respective responses to TNFI and IL-6RI because each sample size was small. Further studies in a large-scale cohort are required to fully determine the role of anti-PPAD immunity on the efficacy of TNFI and IL-6RI therapy. Second, retrospective cohort studies are associated with a selection bias. The mean age of participants and the percentage of female participants in the present study were similar to those in another large-scale race-matched cohort study [44], which suggests that the selection bias was minimized. However, the possibility of a selection bias cannot be excluded. Third, the lack of a control group cannot provide certain information, such as whether patients with baseline low anti-PPAD IgG titers would respond to csDMARD treatment as well. However, it was difficult ethically to monitor control patients with RA and periodontitis who only received csDMARD without periodontal therapy for 6 months, because the therapeutic effects of these drugs on periodontitis have not yet been clarified [6]. Finally, the clinical response was assessed after 3 and 6 months of bDMARD therapy according to the proposal of a concise report [45]. However, caution is necessary when the interpreting the present data due to the long period of time since the patients were initially diagnosed with RA. It might be promising to evaluate the response for a longer period from soon after RA diagnosis to determine a reliable predictor for the efficacy of bDMARD therapy. Despite these limitations, the present study is unique in that parallel evaluations were performed by professional rheumatologists and periodontists to minimize biases in the data collection.

In conclusion, the results of the present study showed that the serum IgG levels to PPAD affect the clinical response to bDMARD in patients with RA. Determination of the serum anti-PPAD IgG titers might help in designing a new personalized treatment strategy for RA.
